# Increasing the biosafety of analytical systems in the clinical laboratory

**DOI:** 10.1155/S1463924694000076

**Published:** 1994

**Authors:** A. Truchaud, P. Schnipelsky, H. L. Pardue, J. Place, K. Ozawa

**Affiliations:** 1 Institut de Biologie de Hopitaux de Nantes BP 1005 Nantes Cedex 44035 France

## Abstract

Biosafety is an important part of the know-how of all clinical laboratory professionals. Biosafely must have high priority in the design and use of analytical systems. Attention should be focused on reducing the handling of biological specimens, reducing biohazards to laboratory personnel, and on improving the labelling and containment of biohazardous materials. In this paper, biosafety issues are discussed in relation to the design of analytical systems, their use and maintenance.


Journal of Automatic Chemistry, Vol. 16, No. 2 (March–April 1994), pp. 67–70

### Increasing the biosafety of analytical systems in the clinical laboratory

**Prepared for the International Federation of Clinical Chemistry by A. Truchaud*, P. Schnipelsky, H. L. Pardue, J. Place and K. Ozawa**

*Biosafety is an important part of the know-how of all clinical laboratory professionals. Biosafety must have high priority in the design and use of analytical systems. Attention should be focused on reducing the handling of biological specimens, reducing biohazards to laboratory personnel, and on improving the labelling and containment of biohazardous materials. In this paper, biosafety issues are discussed in relation to the design of analytical systems, their use and maintenance.*

### Introduction

Biological materials, particularly blood and urine, may present health risks to laboratory workers. These risks are dramatically increased when infectious agents, such as the hepatitis viruses and HIV, are present [1, 2]. Other compounds, such as carcinogens, toxic organic compounds, teratogens and some metals, that are present in reagents also pose biosafety hazards. A number of organizations have produced guidelines which are focused on general rules for laboratory organization and handling of specimens [3–5].

A major recent development in clinical laboratories is the increasing use of robotics in automated analytical systems. An intelligent approach is to take biosafety into account, from the start, in developing new systems. Biosafety should be a system requirement like precision, accuracy or throughput [6, 7]. The purpose of this paper is not to produce detailed specifications; but to demonstrate the advantages which would result from efforts in this area.

### Specimen collection, labelling and transportation

Biosafety is important in every aspect of laboratory work (see figure 1). Precautions should begin with specimen collection; these have been extensively discussed in the literature [3, 4]. The problems of specimen collection, especially those of specimen containers, should be considered an integral part of the analytical system. Most of the existing specimen collection devices were developed independently of the analytical systems. The following highlights some of their defects and suggests improvements.

Open syringes are potentially dangerous. Closed blood specimen collection systems, such as evacuated sampling devices and tubes, should be used to collect blood samples. A systematic approach linking the sample collection step to analysis of the sample requires that the collection tube be compatible with the analyser. Many analytical systems can take a sample directly from primary tubes. Standardization of specimen collection tubes is necessary to reduce the number of different sizes employed on different types of instrument carousels.

New technologies that take biosafety into consideration are needed. Containers should be designed for collection of specimens other than blood, such as urine and stool, and for their safe transport and handling. Procedures for avoiding infection from cerebrospinal fluid and other types of body fluids, and from stool, should be specified and followed.

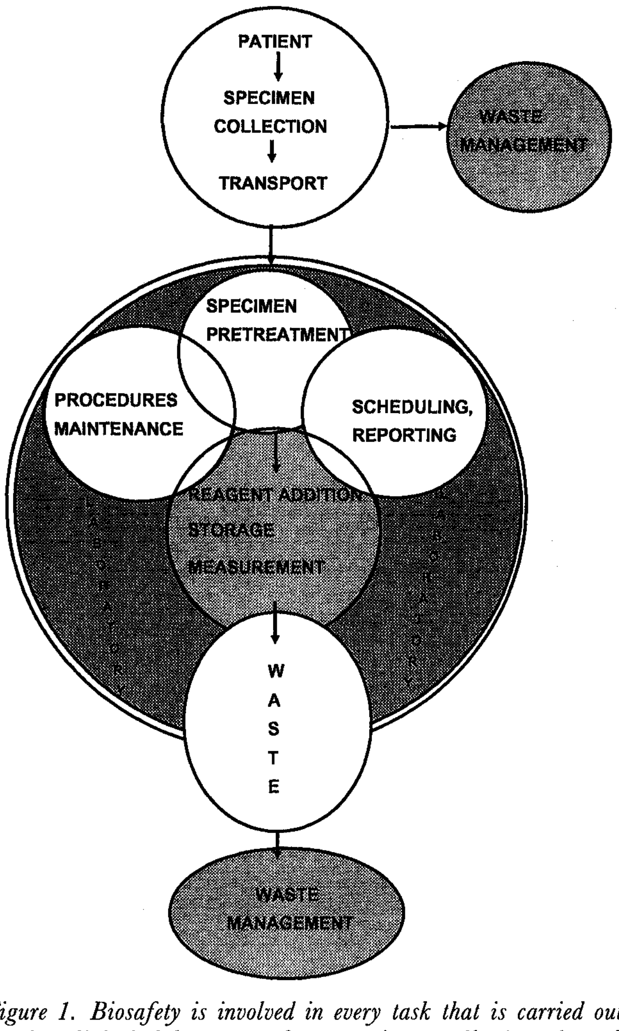

*Figure 1. Biosafety is involved in every task that is carried out in the clinical laboratory, from specimen collection through maintenance and waste disposal.*

* *Correspondence to Professor A. Truchaud, Institut de Biologie de Hopitaux de Nantes, BP 1005, 44035 Nantes Cedex, France. The authors listed above make up the IFCC Committee on Analytical Systems.*

© 1994 IFCC

*Table 1. Biosafety in the handling of whole blood.*

| Step | Recommended action |
|---|---|
| Centrifugation of blood | —Use tubes of a size and external diameter that exactly fits the buckets<br>—Use plastic tubes to reduce breakage and for ease of disposal<br>—Use clear bucket covers to allow inspection of contents |
| Centrifugation of microhaematocrit tubes | —Seal the tubes to avoid aerosol formation<br>—Use a sealed rotor |
| Mixing of samples for blood gas analysis | —Use plastic (instead of glass) capillaries<br>—Use a robotic mixer [16]<br>—Use an arterial blood collection kit[a] |

[a]Such kits are produced by a variety of manufacturers, such as Radiometer, Becton Dickinson, Terumo and Ciba-Corning.

As for labelling of specimens, some guidelines recommend a special label indicating a high risk of infection for some specimens. All specimens should be treated as potentially hazardous [8]. Biohazards are decreased if the amount of handling the specimen undergoes is reduced. To this end, a reliable identification system helps. The labels must have one kind of identification that can easily be read by eye. If possible, identification that can be read by a machine should also be attached to the specimen at the time of collection [9]. Machine-readable labels must be compatible with the sample code reader of the analytical system. Again, it would be useful to standardize the codes employed by different analytical systems.

The main risks in specimen transport are breakage of the specimen container, leakage of its contents due to poor sealing, and partial or complete loss of the specimen. Standardization of the container specifications would allow a standardization of transport devices [5]. A new trend within some hospitals is to mechanize specimen transport to the laboratory using closed containers which can be thermally controlled.

### Preanalytical treatment of blood specimens

In order to separate plasma or serum, some analytical systems include an integrated separation step using filtration or centrifugation [10–12]. However, the technique most often used for large batches of specimens is centrifugation without any special devices.

Table 1 lists the steps that may be taken to reduce the risk of contamination from processing blood. The main risk in centrifugation and in mixing whole blood is contamination by aerosol from open and broken tubes. When whole bood has to be mixed, the risk lies in breakage of the specimen containers and injury to the operator, resulting in infection. These hazards can be avoided once they are identified.

An attractive innovation is the concept of automated serum or plasma processing station, in which centrifugation and transfer of specimen into closed samples cups and secondary storage tubes are automated [13]. It is possible to automate not only the separation process but the whole sample handling process [14].

In blood cell counting, the contents of the tube require mixing before dilution for counting. The operator holds the two ends of the tube between his or her thumb and index finger. The use of the analytical systems for haematology which have built-in automated mixing devices is desirable because it avoids this biohazard. Blood smears for microscopic examination should be prepared robotically for the same reason [15].

### Specimen sampling

In order to avoid transfers of blood, plasma or serum, which always pose a biohazard, specimens should be sampled from the primary tubes. Blood counting systems now have sampling modules that are able to withdraw the specimen from a closed tube through a probe which penetrates the cap. Some chemistry systems are able to withdraw a plasma or serum specimen through a tube that not only penetrates the cap but senses the surface of the fluid specimen [17, 18]. This, or an equivalent approach avoiding manual transfers, should become a standard feature for automated systems.

The sampling probes used in analysers, which are sharp and move at high speed in the carousel area, may pose a hazard to the operator. Probes should be equipped with detectors that stop the process in the case of an obstruction (which is already the case on some robotic systems), and the operator should be 'locked out' of some areas of the analyser when the probe is in operation.

One option is the continuous input of specimens in racks which are automatically moved to the sample area [19]. Ideally, all analysers should use the same form of sample racks which would again reduce the need for transfer of blood tubes. In addition the system for holding the individual samples should be common from the point of reception to the point of disposal or storage of the analysed samples.

To avoid carry-over effects, some analysers use disposable tips which are affixed by a robotic arm and discarded after sample delivery. Aerosol contamination is possible in such automated systems because this type of pipettor uses air displacement to eject used tips, contaminating the pipettor itself. This biohazard can be avoided with the use of pipette tips manufactured with plugs that serve as aeorosol barriers.

In bacteriology, some automated systems for blood cultures allow the cultures to remain closed. Whole bood is introduced into the container through the cap, and gas samples are aspirated through the cap and transferred for $CO_2$ measurement. Recently, an automated colorimetric microbial detection system was described using a $CO_2$ detection system built into the bottom of each bottle and separated from the culture medium by a semipermeable membrane. As $CO_2$ is produced it crosses the membrane. The decrease in pH causes a colour change which is photometrically measured without the need to open the culture bottle [20]. Handling steps can be further reduced in another automated blood culture system in which the $CO_2$ is measured within the specimen container by means of infra-red spectrometry (ARGOS Blood Culture Automated System, Pasteur Diagnostics, France). As a further example, an erythrocyte sedimentation reader has been developed where a video image processing system scans the erythrocyte sedimentation process directly through a dedicated tube [21].

### Biohazards in reagents and quality-control materials

Reagents used in the clinical laboratory may pose a biohazard. For example, the ethidium bromide that is used for staining DNA in cytofluorometry is potentially carcinogenic to the operator. Quality-control and calibration materials must be free of known infectious agents. However, no guarantee can be given that they are free of any infectious agents. Precautions against infectious agents not yet recognized, or not yet detectable, have to be taken into consideration by both the supplier and user of these products. If possible, materials derived from human blood should be avoided.

Several practical points should be made. Quality-control materials and reagents that are supplied in sealed vials requiring sharp instruments (such as scissors) for opening them should be avoided. Second, refrigeration of reagents remaining on the analyser is often desirable because it reduces microbial growth. Third, recycling of reagents, which is possible in some systems, should be avoided because it leads to a high risk of contaminating the system with infectious agents. Systems should be designed to make recycling of reagents impossible.

### Storage and handling of specimens and reagents

When cold storage rooms, refrigerators or freezers are used for storage of specimens or reagents, they should be equipped with separate compartments for specimens and reagents. The specimen areas should be carefully disinfected.

When uncapped cups are used on carousels, it is often difficult to remove them without spilling the serum or plasma. Commonly used anti-evaporation barriers may limit this biohazard.

### Waste disposal

Currently, specimens or reaction mixtures from analysers go down the drain like waste water. Several alternatives are being developed. By sealing disposable cuvettes after use [17], disposal of liquid waste becomes similar to solid waste disposal. Liquids can be treated with disinfectant before disposal, or collected in containers which are then safely disposed of [22]. Sensors should be used to prevent overflow of the containers, which should be located in a place with limited risk of contaminating either the analyser or the operator. Further, liquid waste containers should be designed in a way that prevents contact between the waste and the operator's hands.

Solid waste is produced by analytical systems that use reagents on solid supports (strips, films, coated microtitre plates) or disposable reagent containers. Solid waste can block an analyser mechanically, resulting in contamination of the analyser with patient sera. We suggest that solid waste should fall into containers with a sensor to detect when they are full.

Techniques for the recovery of materials from waste should be developed in order to encourage biosafety. Manufacturers should provide precise information not only on how to decontaminate, but how to treat the waste (by crushing, burning, etc.) in a manner that will allow recovery of the raw materials. This type of conservation would balance the high cost of improving biosafety. Contaminated materials create special challenges in this regard.

### Maintenance procedures and decontamination

Preventive maintenance has a role in biosafety [23]. Cleaning and disinfection procedures should be included and clearly explained in users' manuals [24]. These steps could be incorporated into the system software, rather like a diagnostic program. The components of the system should be decontaminated (such as reagents, samples, used tubes, seal, membranes and filters) should be identified and the appropriate disposal methods should be given.

Field service engineers should be reminded that sharp tools must not be used in disassembling parts which are in contact with blood or blood derivative.

The non-disposable mechanical parts involved in fluid handling (diluter valves, pump cylinders, heaters etc.) should be designed so that they do not come into contact with the specimen. If the part has to come into contact with possibly contaminated fluids, a disposable part should be designed. A particularly good example is the disposable septum which replaces the valve to aspirate alternatively specimen, calibrants or buffers in a blood gas analyser [25].

Decontamination is performed in a machine used in histopathology for cutting frozen sections [26]. Analogous systems could well be developed to other clinical laboratory instruments to reduce biohazards for the operator.

### Biosafety assurance

The design of analytical systems can improve biosafety. Table 2 summarizes several of the features which have as their objectives protection of instrument operators and service personnel. The small objective is to reduce the possibility of contamination and aerosol formation from fluid system leaks.

Many protocols have been published concerning the analytical performance of instruments and automated systems. New protocols concerning biosafety aspects should be developed; for example, a dynamic microbiological test was applied to the evaluation of sealed containers for use in centrifuges [27]. Other protocols should be suggested by manufacturers.

*Table 2. Improving biosafety through instrument design.*

| Feature | Objectives and recommended action |
|---|---|
| Metal or plastic shield | —To isolate the electronic, fluidic and analytical areas and components<br>—To reduce aerosol formation and other forms of contamination |
| Connectors | —Use of quick release, self-sealing plastic tubing for fluid lines, especially waste disposal lines |
| Keyboards | —Designed for use by the instrument operator, they should be protected, flat and easy to clean<br>—Alternatively, keyboards may be replaced by tactile screens, which are easy to clean |
| Air flow | —Use of separate cooling fan or filtered air flow for power supply and circuit board<br>—Flow must not be directed through the sample handling area before reaching other areas<br>—Flow from instrument must not be directed at the operator |
| Vacuum and pressure systems | —Lines used to withdraw biohazardous waste must be equipped with hydrophobic filters located between the pump and the container |
| Liquid drip tray | —When possible, sample handling and probe washing areas should be equipped with plastic tray with drainage into a waste container |
| Biohazard labels | —Instrument parts that may come into contact with patient specimens or waste should be labelled with the internationally recognized biohazard symbol |

The training programme for the laboratory team should include instruction on biosafety, as well as productivity and the clinical relevance of the results. There should be a systematic approach to biosafety and this should be considered when selecting equipment or introducing a new test procedure.

### References

1. Petithory, J. C., *Medecine et Malades Infectieuses* (**1988**), 118.
2. Vlamov, D. and Polk, B. F., *Occupational Medicine*, **2** (1987), 429.
3. US Department of Health and Human Services, *Laboratory Medicine*, **2** (1988), 88.
4. National Committee for Clinical Laboratory Standards (NCCLS), Protection of laboratory workers from infectious disease transmitted by blood and tissue, NCCLS Document M-29-T2 (199). Requests for copies should be addressed to NCCLS, 771 East Lancaster Avenue, Villanova, Pennsylvania 19085, USA.
5. Killander, J., Chippaux, A., Eder, G. *et al.*, Protection of laboratory workers from infectious agents transmitted by blood and other biological material, European Committee for Clinical Laboratory Standards (ECCLS) (1989). Requests for copies should be addressed to ECCLS, University Hospital, S-221 85, Lund, Sweden.
6. Dale, G., *Biochimica Clinica*, **13** (1989), 1341.
7. Grafmeyer, D., Manchon, M., Gouget, B. *et al.*, *Inf. Scient. Biol.*, **16** (1990), 87.
8. Valenti, W. M., *Medical Laboratory Observer*, **2** (1986), 53.
9. Bonini, P. A., *Biochimica Clinica*, **13** (1989), 1331.
10. Burtis, C. A., Johnson, W. F. and Walker, W. A., *Clinical Chemistry*, **32** (1989), 1642.
11. Shultz, S. G., Holen, J. T., Donohue, J. P. and Francoeur, T. A., *Clinical Chemistry*, **31** (1985), 1457.
12. Truchaud, A., Gourmelin, Y., Simon, J. P. *et al.*, *Clinical Chemistry*, **33** (1987), 1560.
13. Maguire, L. C., Cloyd, W. C. and Garrison, T. W., In Program and Abstracts of Oak Ridge Conference on Advanced Analytical Concepts for the Clinical Laboratory (1990); Paper No. 9.
14. Ikeda, T. and Tanaka, F., *Hitachi Review*, **4** (1992), 167.
15. Hayachi, M. and Okamoto, K., *Sysmex Journal*, **13** (1990), 354.
16. Felder, R. A., Margrey, K. S., Boyd, J. C. and Saver, J., *Clinical Chemistry*, **32** (1986) 1103.
17. Driscoll, R. C., Edwards, R. B., Liston, M. D. *et al.*, *Clinical Chemistry*, **29** (1983), 1609.
18. Lipscomb, J. H., Davis, J. E. and Nuzzaci, E. A., In Program and Abstracts of Oak Ridge Conference on Advanced Analytical Concepts for the Clinical Laboratory (1990); Paper No. 36.
19. Alpert, N. L., *Clinical Instrument Systems*, **1** (1989), 1.
20. Thorpe, T. C., Wilson, M. L., Turner, J. E. *et al.*, *Journal of Clinical Microbiology*, **28** (1990), 1608.
21. Patton, W. N., Meyer, P. J. and Stuart, J., *Journal of Clinical Pathology*, **42** (1989), 313.
22. Working Party on the Prevention of Infection (WIP), Prevention of HIV infection in laboratories. Copies are available by writing to Professor R. P. Mouton, Medical Microbiology Laboratory, University Hospital, Building 1-L4-P, Postbus 9600, 2300 RC Leyden, the Netherlands.
23. Truchaud, A., Gouget, B., Gormelin, Y. *et al.*, *Rev. Eur. Tech. Biomed.*, **2** (1989), 111.
24. Larsen, E. and Holbeck, C., Decontamination of ABL blood gas analysers, *Radiometer Copenhagen Document* (1990).
25. Johnson, M. T., Savory, J. and Wills, M. R., *American Clinical Product Review*, (October 1986).
26. Lasslet, A., *European Clinical Laboratory*, (September 1991).
27. Harper, G. J., *Journal of Clinical Pathology*, **37** (1984), 1134.

